# Proton Pump Inhibitors and Serum Magnesium Levels in Patients With Torsades de Pointes

**DOI:** 10.3389/fphar.2018.00363

**Published:** 2018-04-20

**Authors:** Pietro E. Lazzerini, Iacopo Bertolozzi, Francesco Finizola, Maurizio Acampa, Mariarita Natale, Francesca Vanni, Rosella Fulceri, Alessandra Gamberucci, Marco Rossi, Beatrice Giabbani, Michele Caselli, Ilaria Lamberti, Gabriele Cevenini, Franco Laghi-Pasini, Pier L. Capecchi

**Affiliations:** ^1^Department of Medical Sciences, Surgery and Neurosciences, University of Siena, Siena, Italy; ^2^Cardiology Intensive Therapy Unit, Department of Internal Medicine, Hospital of Carrara, Carrara, Italy; ^3^Stroke Unit, University Hospital of Siena, Siena, Italy; ^4^Department of Molecular and Developmental Medicine, University of Siena, Siena, Italy; ^5^Centre of Pharmacovigilance, University Hospital of Siena, Siena, Italy; ^6^Department of Medical Biotechnologies, University of Siena, Siena, Italy

**Keywords:** proton-pump inhibitors, Torsades de pointes, serum magnesium levels, long-QT syndrome, sudden cardiac death

## Abstract

**Background:** Torsades de pointes (TdP) is a life-threatening ventricular tachycardia occurring in long QT-syndrome patients. It usually develops when multiple QT-prolonging factors are concomitantly present, more frequently drugs and electrolyte imbalances. Since proton–pump inhibitors (PPIs)-associated hypomagnesemia is an increasingly recognized adverse event, PPIs were recently included in the list of drugs with conditional risk of TdP, despite only few cases of TdP in PPI users have been reported so far.

**Objectives:** Aim of the present study is to evaluate whether PPI-induced hypomagnesemia actually has a significant clinical impact on the risk of TdP in the general population.

**Methods:** Forty-eight unselected patients who experienced TdP were consecutively enrolled (2008-2017). Shortly after the first TdP episode, in those patients who did not receive magnesium sulfate and/or potassium or calcium replacement therapy, serum electrolytes were measured and their relationship with PPI usage analyzed.

**Results:** Many patients (28/48, 58%) were under current PPI treatment when TdP occurred. Among TdP patients in whom serum electrolyte determinations were obtained before replacement therapy (27/48), those taking PPIs had significantly lower serum magnesium levels than those who did not. Hypomagnesemia occurred in ~40% of patients receiving PPIs (6/14), in all cases after an extended treatment (>2 weeks). In patients taking PPIs the mean QT-prolonging risk factor number was significantly higher than in those who did not, a difference which was mainly driven by lower magnesium levels.

**Conclusions:** In unselected TdP patients, PPI-induced hypomagnesemia was common and significantly contributed to their cumulative arrhythmic risk. By providing clinical support to current recommendations, our data confirm that more awareness is needed when a PPI is prescribed, specifically as regards the risk of life-threatening arrhythmias.

## Introduction

Torsades de pointes (TdP) is a life-threatening polymorphic ventricular tachycardia that can degenerate into ventricular fibrillation (VF) and cause sudden cardiac death (SCD) (Drew et al., 2010). It is characterized by a pattern of twisting points and occurs in patients with long QT syndrome (LQTS), both acquired and congenital. Indeed, in congenital-LQTS the more the heart rate-corrected QT interval (QTc) prolongs, the greater the TdP risk exponentially increases (i.e., 5–7% risk increase each 10 ms prolongation in QTc) until being significant for QTc>500 ms; such a value associated with a 2–3-fold higher risk for TdP (Drew et al., 2010).

Since a marked QTc prolongation is usually required for TdP development, in most cases the simultaneous presence of multiple QTc-prolonging factors synergistically operating in impairing ion channels responsible for the ventricular repolarization process is necessary. Congenital factors are included, mainly resulting from mutations affecting genes encoding for potassium or sodium channels, as well as acquired risk factors (Viskin, 1999; El-Sherif and Turitto, 2003; Drew et al., 2010; Itoh et al., 2016). Among the latter factors, electrolyte imbalances (i.e., hypokaliemia, hypocalcemia, hypomagnesemia) and QT-prolonging drugs blocking the hERG potassium channel are those most frequently implicated in TdP development. Other established causes of acquired LQTS and TdP include structural heart diseases, bradyarrhythmias, endocrine disorders, liver diseases, nervous system injuries, HIV infection, starvation, hypothermia and toxins (El-Sherif and Turitto, 2003; Drew et al., 2010). In addition, autoimmunity (Lazzerini et al., 2017d) (particularly anti-Ro/SSA antibodies) (Yue et al., 2015; Lazzerini et al., 2016) and systemic inflammation (Lazzerini et al., 2015, 2017a,b) in the recent years are being increasingly recognized as novel acquired QT-prolonging risk factors significantly impacting TdP risk in the general population.

Proton–pump inhibitors (PPIs) are the most effective therapeutic agents for acid related disorders (ARD), including peptic ulcer disease and gastroesophageal reflux disease (Strand et al., 2017). Moreover, such drugs are also used for the prevention of non-steroidal anti-inflammatory drug-induced gastric injury and as a part of *Helicobacter pylori* eradication regimens (Strand et al., 2017). As a result, PPIs currently represent the fifth best-selling drug in the market with millions of chronic users worldwide (Patterson Burdsall et al., 2013). During the last years, concern has been raised because of PPIs long-term overutilization. In fact, in the clinical practice PPIs are often prescribed in patients without a specific ARD, and such a habit is leading to significant cost expenditure and possible adverse events (Moayyedi and Leontiadis, 2012).

Hypomagnesemia is a potentially serious side effect of PPIs, that could account for ~1% of all adverse events reported by drug users (Famularo et al., 2013; Luk et al., 2013). Although several data suggest an interference on intestinal magnesium absorption, the exact underlying mechanism is poorly understood (Famularo et al., 2013). In 2011 the US FDA warned that long-term use of PPI has the potential to reduce circulating magnesium levels, particularly in patients concomitantly receiving other drugs capable to cause magnesium depletion such as diuretics (2011)^1^. Accordingly, in 2016 the Arizona Center for Education and research on Therapeutics (AZCERT) included the PPIs omeprazole, esomeprazole, lansoprazole and pantoprazole in the list of drugs with conditional risk of TdP and to be avoided in patients with congenital LQTS (AZCERT, 2016), despite only few cases of QTc prolongation and TdP have been reported in patients with severe PPI-induced hypomagnesemia and/or taking a PPI concomitantly with drugs known to directly prolong QTc (Asajima et al., 2012; Bibawy et al., 2013; Hansen and Bruserud, 2016). As a result, it is now recommended that in patients taking a PPI for an extended period of time (>2 weeks) serum magnesium levels be monitored periodically, particularly if extended PPI therapy is used in association with drugs carrying a known risk of TdP (Asajima et al., 2012; 2016). Notably, a very recent longitudinal observational study performed in a large primary cohort of new users of acid suppression therapy followed for a median of 5.7 years, found a significant association between PPI use and risk of all-cause mortality. The risk was increased among those with no documented medical indications for PPI use and prolonged duration of use (Xie et al., 2017).

Regardless of official recommendations, available real-life information on this subject is relatively poor so far. The present study is specifically aimed at evaluating whether PPI-induced hypomagnesemia has a significant clinical impact on the risk of TdP in the general population. Thus, the actual usage of PPIs and its relationship with serum magnesium levels were analyzed in a cohort of TdP patients, prospectively and consecutively enrolled independent of ongoing therapies and concomitant diseases.

## Patients and methods

### Study populations

Local Ethical Committee approved the study, and patients gave their oral and written informed consent in accordance with the Principles of the Declaration of Helsinki.

We prospectively enrolled (from January 2008 to May 2017) 48 consecutive hospitalized patients who presented with TdP, independent of ongoing therapies and concomitant diseases. Since the only inclusion criteria was the occurrence of TdP, all patients who came to our attention in that period of time were enrolled. No patients were excluded. Demographic, clinical and laboratory characteristics of study patients, as well as ongoing treatment with QTc-prolonging medications are provided in Table 1. In these patients, PPI usage was assessed, and a cut-off time of 2 weeks was used to define treatment duration as extended (>2 weeks) or not, according to current AZCERT recommendations to minimize the risk of TdP in patients treated with PPI (AZCERT, 2016).

**Table 1:** Demographic, clinical and laboratory characteristics of patients with Torsades de pointes.

**Patients, n**	**48**
Age, median years (interquartile range)	81(73–85)
Females, n	31/48(65%)
Mean QTc, ms(range)	596.0 ± 80.7(490–910)
Electrolyte imbalances, n	37/47(79%)
Hypokaliemia	28/45(62%)
Hypocalcemia	22/37(59%)
Hypomagnesemia	7/27(26%)
Concomitant diseases^*^, n	45/48(94%)
*Cardiac diseases*	40/48(83%)
Left ventricular hypertrophy	19/48(40%)
Dilated cardiomyopathy/heart failure	13/48(27%)
II-III degree atrioventricular block	10/48(21%)
Acute coronary syndrome	9/48(19%)
Chronic coronary artery disease	7/48(15%)
Sinus bradycardia	6/48(13%)
*Extra-cardiac diseases*	20/48(42%)
Diabetes mellitus type II	13/48(27%)
Chronic kidney disease	8/48(17%)
Hypothyroidism	2/48(4%)
Subarachnoid hemorrhage	1/48(2%)
Cirrhosis	1/48(2%)
Anorexia nervosa	1/48(2%)
HIV infection	1/48(2%)
QTc prolonging-medications, n	34/48(71%)
Amiodarone	14/48(29%)
Citalopram	5/48(10%)
Sertraline	4/48(8%)
Fluconazole	3/48(6%)
Trazodone	3/48(6%)
Levofloxacin	2/48(4%)
Clarithromycin	2/48(4%)
Promazine	2/48(4%)
Quetiapine	2/48(4%)
Mean medication number per patient	1.1 ± 1.0
Anti-Ro/SSA positivity, n	18/32(56%)
Systemic inflammation, n^†^	38/48(79%)
C-reactive protein, mg/dl(range)	2.66(0.1–29.65)
Definite inflammatory diseases	22/48(46%)
Acute infections	15/48(31%)
Immuno-mediated diseases	5/48(10%)
Others	2/48(4%)
Mean QTc-prolonging risk factor number per patient^§^	5.3 ± 1.5

*Except where indicated otherwise, data are expressed as mean ± standard deviation or median (range)*.

*Appropriate serum potassium, calcium or magnesium measurements available in 45, 37, and 27 out of 48 patients, respectively; anti-Ro/SSA antibodies tested in 32 out of 48 patients*.

* *Diseases recognized to be a risk factor for QTc prolongation (Viskin, 1999; El-Sherif and Turitto, 2003; Drew et al., 2010)*.

† *Increased C-reactive protein level (>0.5 mg/dl) with or without a definite inflammatory disease*.

§ *Including electrolyte imbalances, diseases, QTc-prolonging medications, anti-Ro/SSA positivity, and systemic inflammation (Viskin, 1999; El-Sherif and Turitto, 2003; Drew et al., 2010; Yue et al., 2015; Lazzerini et al., 2016, 2017b)*.

### ECG recordings

**Figure 1:**
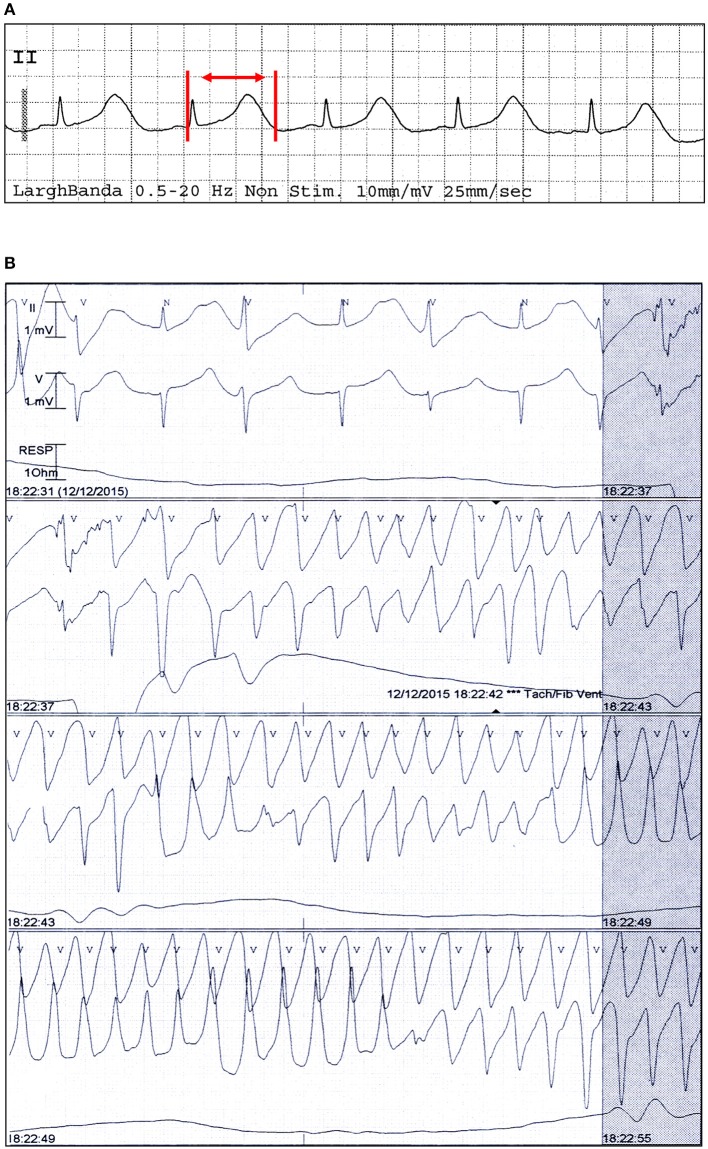
Electrocardiographic findings of a patient with TdP and PPI-associated hypomagnesemia. ECG strip in sinus rhythm **(A)** and during TdP **(B)** from a patient who was under current and extended treatment with oral lansoprazole (15 mg/day), and had low magnesium levels (1.46 mg/dl) and a QTc of 670 ms. Red vertical lines and arrow in lead II show QT interval.

Diagnosis of TdP was based on the presence of at least one episode of polymorphic ventricular arrhythmia at a rate ranging from 160 to 240 beats/min, associated with QTc prolongation (Drew et al., 2010; Figure 1). The QT interval was manually measured on a standard 12-lead ECG, from the onset of the Q wave or the onset of the QRS complex to the end of the T wave, defined as the return to the T-P baseline. When present, prominent U waves (>1 mm) merging into T waves were included in QT measurement (Gupta et al., 2007). QTc, determined as the longest hand-measured QTc in any lead (Rautaharju et al., 2009) was corrected for heart rate by the Bazett formula (dividing the QT by the square root of the preceding R-R interval of each beat: QT/√RR) to yield the QTc value. QTc was measured from 3 non-consecutive beats (mean value) by a single investigator.

### Laboratory analysis

Shortly after the first TdP episode [no later than 24 h (median 6 h, range 1–22 h)], patients underwent a venous withdrawal to determine serum electrolyte levels, including potassium, sodium, calcium, and magnesium. Potassium and sodium were determined by indirect potentiometry (COBAS-6000 platform); values were expressed as mEq/L (reference values: potassium 3.5–5.5; sodium 132–148). Calcium and magnesium were assayed by a colorimetric method (COBAS-6000 platform); values were expressed as mg/dl (reference values: calcium 8.0-11.0; magnesium 1.5–2.5).

Only determinations obtained before the administration of intravenous magnesium sulfate and/or replacement therapy with potassium or calcium were considered appropriate to be included in the study. As a result, serum potassium, calcium or magnesium measurements were available in 45, 37, and 27 out of 48 patients, respectively.

Other laboratory parameters included circulating levels of anti-Ro/SSA antibodies (*see* Supplementary Methods for more details) and C-reactive protein (CRP), as well as pH, bicarbonates and serum glucose.

### Statistical analysis

To compare TdP patients subgroups, the following parametric or non-parametric statistical analyses were respectively carried out: the two-tail Student's unpaired *t*-test, or the two-tail Mann-Whitney test to evaluate differences in quantitative variables; the Pearson or Spearman rank correlation-test to verify possible statistical association between quantitative variables; the two-sided Fisher's exact test to evaluate statistical correlation between categorical variables. *p* < 0.05 were considered as significant. All statistical analyses were performed using GraphPad-InStat, version 3.06 for Windows 2000.

## Results

### TdP patients characteristics

As detailed in Table 1, demographic, clinical and laboratory characteristics of our cohort were fully consistent with those expected in TdP patients based on established epidemiological data. In fact, the large majority of subjects were females (31/48, 65%) and older than 65 years (median age: ~80 years). Moreover, many recognized QTc-prolonging risk factors of acquired origin were identifiable, particularly an underlying cardiac disease (45/48, 83%, more frequently ventricular hypertrophy, dilated cardiomyopathy/heart failure and atrio-ventricular blocks), electrolyte imbalances (37/47, 79%) and QTc-prolonging medications (34/48, 71%). Hypokalemia occurred in 62% of patients (28/45), thereby representing the most common specific risk factor. Anti-Ro/SSA-52 kD antibodies were detected in 56% of the tested cases (18/32), although a history of autoimmune disease was present in two patients only (1 rheumatoid arthritis, 1 celiac disease). The majority of TdP patients (38/48, 79%) showed signs of systemic inflammation, as indicated by the increase in CRP levels (>0.5 mg/dl; median value 2.66 mg/dl). A definite inflammatory disease was present in 22/48 patients (46%), most commonly an acute infection (*n* = 15, particularly sepsis and pneumonia), but also chronic immune-mediated diseases (*n* = 5, including 3 chronic inflammatory arthritis), or acute aseptic inflammatory processes (*n* = 2). Among drugs, amiodarone was the most frequently administered (14/48, 29%). Notably, in almost all cases more than one known QTc-prolonging factor was simultaneously identifiable; on average ~5. In addition, a significant proportion of patients (25/48, 52%) experienced an adverse short-term arrhythmic outcome, i.e., VF/cardiac arrest (CA), and/or underwent electric shock (TdP rapidly degenerated to VF/CA; out-of-hospital VF/CA followed with DC-shock, only later revealing a manifestation of TdP episodes; sustained TdP not responsive to medical therapy).

### Proton-pump inhibitors usage in TdP patients

In our cohort, a significant percentage of patients were under active treatment with PPI when TdP occurred (28/48, 58%). Many subjects (16/25, 64%) were taking a PPI for an extended period of time, i.e., >2 weeks. The most frequently administered PPI was pantoprazole, followed by lansoprazole, together accounting for ~85% of the cases (24/28). Remaining patients (*n* = 4), were administered with omeprazole (*n* = 3), or esomeprazole (*n* = 1). In three patients under extended home PPI therapy, the molecule was changed during hospitalization, before TdP development (from oral lansoprazole or pantoprazole to intravenous pantoprazole in two cases; from oral omeprazole to oral pantoprazole in the other one). The commonest route of administration was the oral one, but in 6/28 cases (21%) where the PPI was being given intravenously at the time of TdP occurrence (Table 2). Notably, none of the intravenously-treated patients showed hypomagnesemia.

**Table 2:** Proton-pump inhibitors use in patients with Torsades de Pointes.

Patients under active treatment with PPIs, n	28/48(58%)
**Specific PPI used, n**	
Pantoprazole	18/28(64%)
Lansoprazole	6/28(21%)
Omeprazole	3/28(11%)
Esomeprazole	1/28(4%)
Treatment duration^*^	
Extended therapy (>2 weeks), n	16/25(64%)
Not extended therapy (<2 weeks), n	9/25(36%)
**Daily dose, mg**^†^	
Pantoprazole	33.3 ± 9.7
Lansoprazole	27.5 ± 6.1
Omeprazole	26.7 ± 11.5
Esomeprazole	20
**Route of administration, n**^†^	
Oral	22/28(79%)
Intravenous	6/28(21%)

*Except where indicated otherwise, values are expressed as mean ± standard deviation*.

* *Data missing in 3 out of 28 patients*.

† *At the moment of TdP occurrence*.

### Serum electrolytes levels and other TdP risk factors in patients taking or not taking proton-pump inhibitors

**Figure 2:**
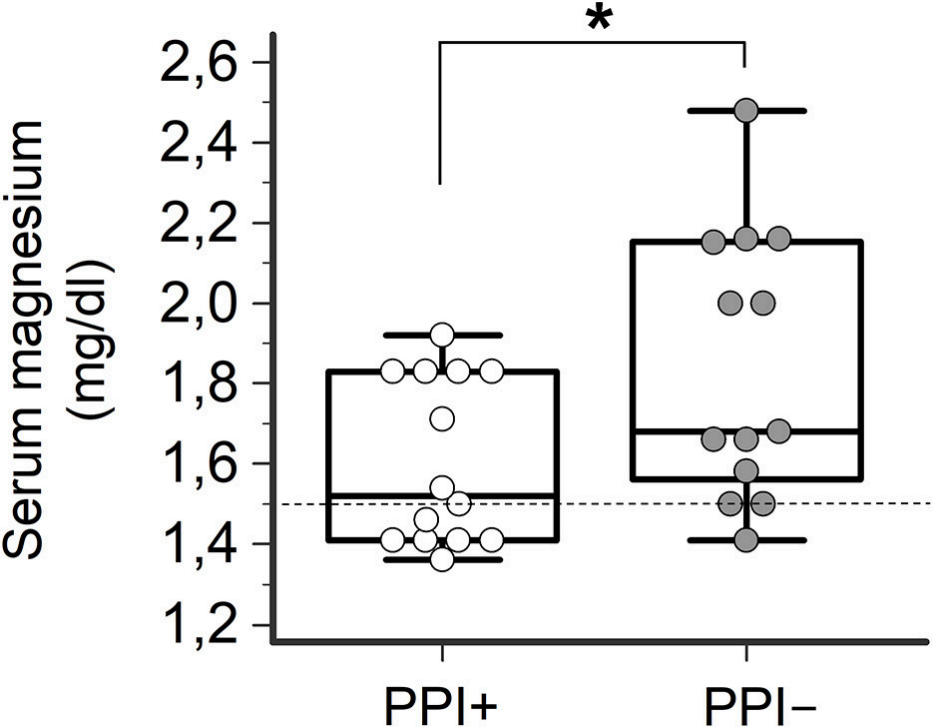
Serum magnesium levels in TdP patients taking or not taking PPIs. Patients taking PPIs (PPI+), *n* = 14; patients not taking PPIs (PPI−), *n* = 13. Two-tail Student's unpaired *t*-test, ^*^p < 0.05. Horizontal dotted line indicates the lower limit of reference values for serum magnesium levels, i.e., 1.5 mg/dl.

**Figure 3:**
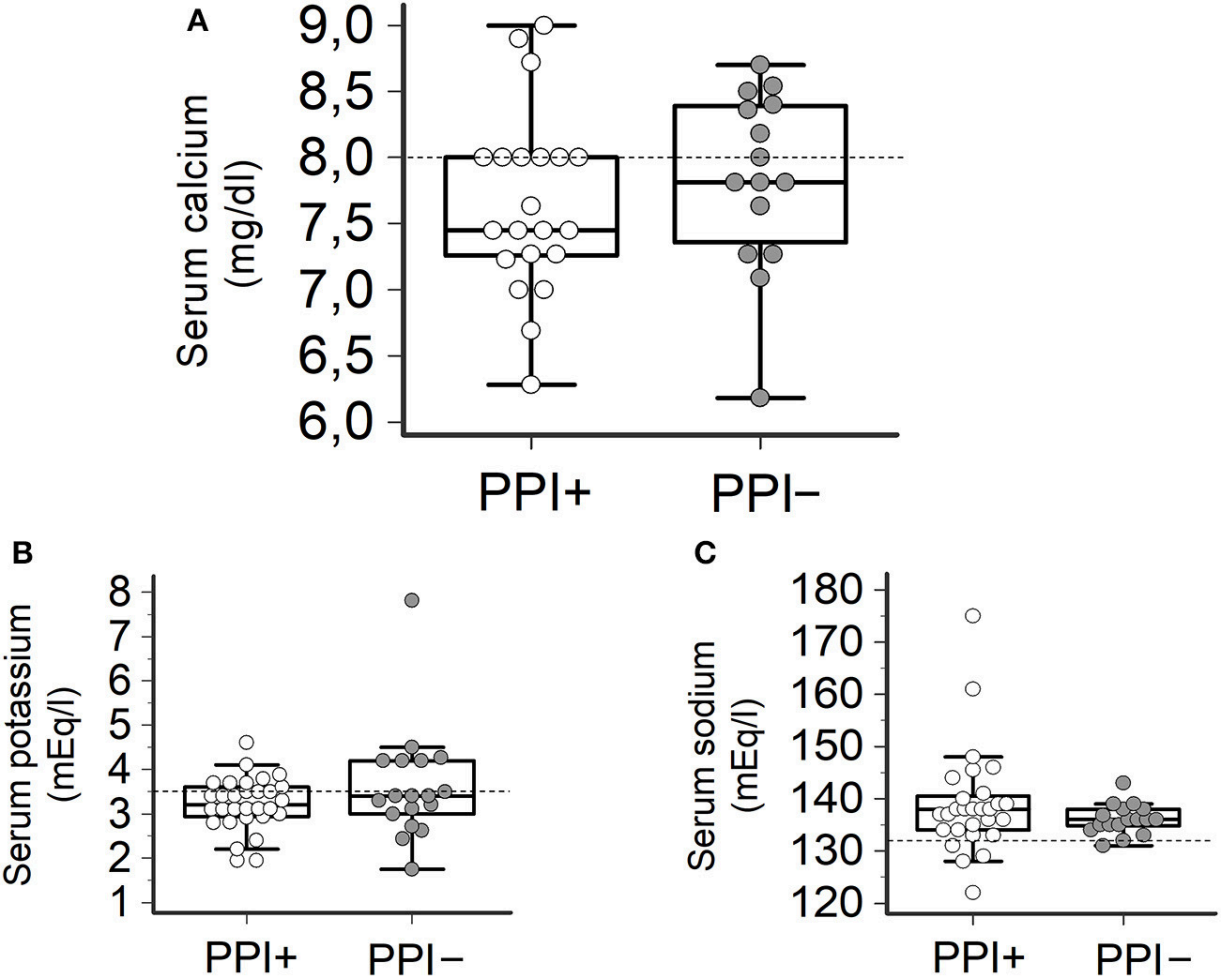
Serum levels of calcium, potassium and sodium in TdP patients taking or not taking PPIs. **(A)** Serum calcium levels. Patients taking PPIs (PPI+), *n* = 20; patients not taking PPIs (PPI−), *n* = 17. Two-tail Student's unpaired *t*-test (*p* > 0.05). Horizontal dotted line indicates the lower limit of reference values for calcium levels, i.e., 8.0 mg/dl. **(B)** Serum potassium levels. PPI+, *n* = 27; PPI−, *n* = 18. Two-tail Student's unpaired *t*-test (*p* > 0.05). Horizontal dotted line indicates the lower limit of reference values for potassium levels, i.e., 3.5 mEq/L. **(C)** Serum sodium levels. PPI+, *n* = 28; PPI−, *n* = 20. Two-tail Student's unpaired *t*-test (*p* > 0.05). Horizontal dotted line indicates the lower limit of reference values for sodium levels, i.e., 132 mEq/L.

Consistently with the findings obtained in the whole TdP population, a high prevalence of electrolyte imbalances (collectively ~80%) was found in both patients taking (PPI+) or not taking PPI (PPI−). However, while the prevalence of hypokaliemia and hypocalcemia as well as serum potassium, calcium (and sodium) levels in the two groups were overalapping, circulating magnesium levels were significantly lower in PPI+ than in PPI− subjects (1.60 ± 0.21 vs. 1.84 ± 0.33 mg/dl, Δ = −0.24 mg/dl; *p* = 0.03) (Figures 2, 3). Hypomagnesemia (< 1.5 mg/dl) occurred 5-times more frequently in the PPI+ vs. PPI- group (6/14, 43% vs. 1/13, 8%), although this difference did not reach statistical significance (*p* = 0.07) (Table 3). Notably, hypomagnesemia was found almost exclusively (6 out of 7 cases, 85%) in patients receiving PPI therapy; all cases of PPI-associated hypomagnesemia (*n* = 6) were observed in patients under extended PPI therapy (>2 weeks), involving all the 4 different PPIs used in the cohort (pantoprazole, *n* = 3; lansoprazole, *n* = 1; omeprazole, *n* = 1; esomeprazole, *n* = 1). Diuretics usage, which was not different in the PPI+ vs. PPI− group (Table 3), was not *per se* associated with significant magnesium changes in our cohort. In fact, by comparing patients taking (*n* = 16) and not taking diuretics (*n* = 11), neither serum magnesium levels (1.67 ± 0.31 vs. 1.78 ± 0.26 mg/dl; *p* = 0.32, two-tail unpaired *t*-test) nor the prevalence of hypomagnesemia (6/16, 37% vs. 1/11, 9%; *p* = 0.18, two-sided Fisher's exact test) were significantly different. Although these findings suggest that diuretics alone, differently to PPIs alone, were not sufficient to cause magnesium depletion, nevertheless diuretics may exacerbate PPI-associated magnesium reduction when administered in association. Indeed, in patients concomitantly receiving PPIs and diuretics (*n* = 9, vs. others *n* = 18) serum magnesium levels further decreased slightly (1.55 ± 0.21 vs. 1.80 ± 0.30 mg/dl, Δ = −0,25 mg/dl; *p* = 0.02, two-tail Mann-Whitney test), and the prevalence of hypomagnesemia increased, reaching statistical significance (5/9, 56% vs. 2/18, 11%; *p* = 0.02, two-sided Fisher's exact test). Despite a specific investigation, no any significant impact of other common causes of hypomagnesemia was found in our cohort of patients (see Supplementary Results for more details). Moreover, no significant correlation was present between magnesium levels and other continuous variables, particularly calcium (*r* = 0.33, *p* = 0.10; Pearson correlation-test) potassium (*r* = 0.10, *p* = 0.57; Pearson correlation-test), sodium (*r* = 0.11, *p* = 0.58; Spearman rank correlation-test) or CRP levels (*r* = −0.14, *p* = 0.46; Spearman rank correlation-test), or QTc duration (*r* = −0.19, *p* = 0.32; Pearson correlation-test).

**Table 3:** Demographic, clinical and laboratory characteristics in proton pump inhibitor users (PPI+) vs. non-proton pump inhibitor users (PPI−).

	**PPI+**	**PPI−**	***p***
Patients, n	28	20	
Age, median years (interquartile range)	80.5(73–85)	81.5(75–87.5)	0.40
Females, n	18/28(64%)	15/20(75%)	0.53
Mean QTc, ms	591.9 ± 88.8	601.5 ± 70.1	0.69
FV/CA/EcS	15/28(54%)	10/20(50%)	1
Electrolyte imbalances, n	22/28(79%)	15/19(79%)	1
Hypokaliemia	17/27(63%)	11/18(61%)	1
Hypocalcemia	12/20(60%)	10/17(59%)	1
Hypomagnesemia	6/14(43%)	1/13(8%)	0.07
Potassium, mEq/L (r.v.3.5–5.5)	3.25 ± 0.61	3.41 ± 0.74	0.47
Calcium, mg/dl (r.v.8.0–11.0)	7.71 ± 0.67	7.85 ± 0.65	0.44
Magnesium, mg/dl (r.v.1.5–2.5)	1.60 ± 0.21	1.84 ± 0.33	**0.03**
Sodium, mEq/L (r.v.132–148)	139.1 ± 10.0	136.1 ± 2.9	0.23
Diuretics use, n	19/28(68%)	10/20(50%)	0.24
Furosemide median daily dose, mg (range)	25(10–100)	72.5(20–500)	0.58
Glucose, mg/dl	171.2 ± 78.8	172.8 ± 80.4	0.96
pH	7.46 ± 0.11	7.50 ± 0.12	0.53
Bicarbonates, mmol/L	25.4 ± 1.8	25.3 ± 2.2	0.97
Concomitant diseases^*^, n	26/28(93%)	19/20(95%)	1
Cardiac diseases	23/28(82%)	17/20(85%)	1
Extra-cardiac diseases	14/28(50%)	6/20(30%)	0.23
QTc prolonging-medications, n	21/28(75%)	13/20(65%)	0.52
Amiodarone	8/28(29%)	6/20(30%)	1
Mean medication number per patient	1.3 ± 1.1	1.0 ± 0.9	0.17
Anti-Ro/SSA positivity, n	8/18(44%)	10/14(71%)	0.16
Systemic inflammation, n	23/28(82%)	13/20(80%)	1
Mean QTc-prolonging risk factor number per patient			
Per patient^†^	5.8 ± 1.6	4.9 ± 1.	**0.04**
Mean QTc-prolonging risk factor number			
Per patient^†^ excluding hypomagnesemia	5.6 ± 1.5	4.9 ± 1.4	0.07

*Wherever not specified, data are expressed as mean±standard deviation. Appropriate serum potassium, calcium or magnesium measurements available in 45, 37, and 27 out of 48 patients, respectively; anti-Ro/SSA antibodies tested in 32 out of 48 patients*.

*VF, ventricular fibrillation; CA, cardiac arrest; EcS, electric shock*.

* *Diseases recognized to be a risk factor for QTc prolongation (Viskin, 1999; El-Sherif and Turitto, 2003; Drew et al., 2010)*.

† *Including electrolyte imbalances, diseases, QTc-prolonging medications, anti-Ro/SSA positivity, and systemic inflammation (Viskin, 1999; El-Sherif and Turitto, 2003; Drew et al., 2010; Yue et al., 2015; Lazzerini et al., 2016, 2017b)*.

*Differences were evaluated by the two-tailed unpaired t-test, or the two-tailed Mann-Whitney test. Difference in categorical variables were evaluated by the two-sided Fisher's exact test*.

*Statistically significant p values are reported in bold*.

As regards the other QTc-prolonging risk factors of acquired origin, individually considered, no significant differences in terms of concomitant diseases, both cardiac and extra.-cardiac, QTc prolonging medications use, anti-Ro/SSA positivity or presence of systemic inflammation were observed by comparing PPI+ vs. PPI− patients (Table 3). Nevertheless, when all these factors were considered together, also including electrolyte imbalances, the mean QTc-prolonging risk factor number per patient was significantly higher in the PPI+ than the PPI- group (5.8 ± 1.6 vs. 4.9 ± 1.4, Δ: 0.9; *p* = 0.04). Notably, statistical significance of this difference was lost if hypomagnesemia, i.e., the only individual TdP risk factor discriminating the two groups, was selectively excluded by the total count (5.6 ± 1.5 vs. 4.9 ± 1.4, Δ: 0.7; *p* = 0.07; Table 3). It is important to underline that for a number of patients, some data on QT-prolonging risk factors were missing, particularly serum levels of potassium (available in 26/28 of PPI+ 19/20 of PPI− patients, respectively), calcium (27/28 of PPI+ and 18/20 of PPI− patients, respectively), magnesium (14/28 of PPI+ and 13/20 of PPI− patients, respectively), and anti-Ro/SSA positivity (18/28 of PPI+ and 14/20 of PPI− patients, respectively). Nevertheless, when we restricted the analysis to patients with full data only, i.e., 8 PPI+ and 10 PPI−, differences (Δ) in mean QTc-prolonging risk factor number per patient remained completely unchanged, both when all risk factors were considered (6.1 ± 1.7 vs. 5.2 ± 1.3, Δ: 0.9) and when hypomagnesemia was excluded (5.8 ± 1.4 vs. 5.1 ± 1.3, Δ: 0.7), thus indicating that the results were not influenced by missing data.

Conversely, PPI treatment did not seem to affect the short-term outcome in our cohort of patients. In fact, the percentage of subjects experiencing VF/CA, and/or that underwent electric shock was not significantly different by comparing PPI+ vs. PPI− patients (15/28, 54% vs. 10/20, 50%) (Table 3).

**Figure 4:**
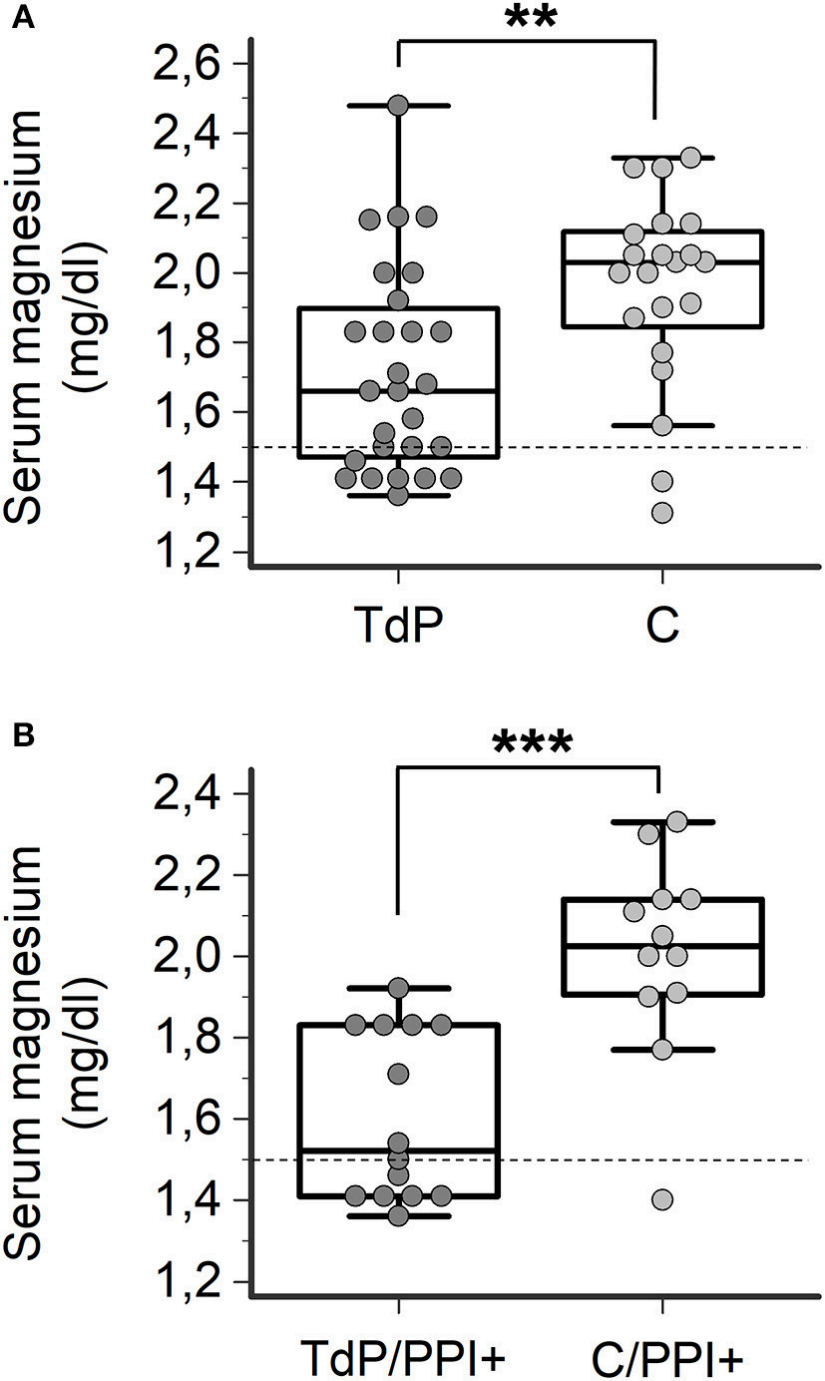
Comparison of serum magnesium levels in TdP patients and controls. **(A)** Serum magnesium levels in all TdP patients (*n* = 27) vs. controls (C, *n* = 21), regardless of PPI therapy. Two-tail Mann-Whitney test, ^**^p < 0.01. (**B**) Serum magnesium levels in TdP patients under PPI therapy (TdP/PPI+) (*n* = 14) vs. controls under PPI therapy (C/PPI+, *n* = 12). Two-tail Student's unpaired *t*-test, ^***^p < 0.001. Horizontal dotted line indicates the lower limit of reference values for magnesium levels, i.e., 1.5 mg/dl.

Finally, in order to specifically address the question of whether magnesium levels are different between PPI+ patients who developed TdP vs. PPI+ patients who did not, 21 hospitalized patients matched for age, gender and concomitant diseases (Supplementary Table 1), but without QTc prolongation or history of TdP were prospectively enrolled as a control group (C). Similarly to that observed in TdP subjects, more than a half of control patients were under current treatment with PPIs (12/21, 57%), in most cases for an extended period of time (10/12, 83%) (Supplementary Table 2). Among these patients, hypomagnesemia was found in 2 patients (2/21, 9%), one treated and one untreated with PPIs. As shown in Figure 4A, circulating magnesium levels were significantly lower in TdP vs. controls (1.72 ± 0.30 vs. 1.91 ± 0.40 mg/dl; *p* = 0.0094). Such a difference significantly increased when the comparison was restricted to PPI-treated patients from the two groups (TdP/PPI+: 1.60 ± 0.21 vs. C/PPI+: 1.93 ± 0.48 mg/dl; *p* = 0.0007; Figure 4B), while serum magnesium levels were not different in PPI-untreated TdP vs. control patients (TdP/PPI−: 1.84 ± 0.33 vs. C/PPI−: 1.88 ± 0.30 mg/dl; *p* = 0.78, two-tail Student's unpaired *t*-test).

As a confirmation of the results on subgroups, we also evaluated the interaction between magnesemia and PPI treatment (PPI+/PPI−), by combining (multiplying) the two variables in the whole population (TdP vs. C). We found that sample differences between TdP and C in such interaction-corrected levels of magnesium were not longer statistically significant (*p* = 0.09, two-tail Mann-Whitney test).

## Discussion

The key findings of the present study are the following: a large proportion of patients (>50%) who developed TdP were under current treatment with a PPI; TdP patients taking PPIs had significantly lower serum magnesium levels with respect to TdP patients not taking PPIs; hypomagnesemia frequently occurred in patients receiving PPIs (~40%, 6/14), in all cases after an extended period of time (>2 weeks) of administration; in subjects taking PPIs the mean QTc-prolonging risk factor number per patient was significantly higher than it was in those not taking PPIs, a difference which was mainly driven by lower magnesium levels.

Magnesium, representing the most abundant intracellular divalent cation, plays a key role in regulating potassium and calcium channels in the heart (Gupta et al., 2007). Experimental studies demonstrated that cytosolic magnesium promotes repolarization of myocardial cells via modulating effects on several potassium currents, including the rapid component of the delayed rectifier potassium current (IKr) and transient outward current (Ito) (Kelepouris et al., 1993; El-Sherif and Turitto, 2011). Moreover, magnesium markedly inhibits the L(long-lasting)-type calcium current (ICaL), possibly as a result of a direct block of the L-type-calcium channel pore by external magnesium or via modification of the activity of protein kinases or phosphoprotein phosphatases (Zhao et al., 2015). ICaL determines the plateau phase thereby critically contributing to action potential duration (APD) (Viskin, 1999; El-Sherif and Turitto, 2003). Moreover, ICaL is the main depolarizing current that generates early after depolarizations (EADs), in turn representing the primary electrophysiological mechanism underlying TdP development (Viskin, 1999; El-Sherif and Turitto, 2003). This supports the fact that hypomagnesemia is a recognized risk factor for QTc prolongation and TdP (Viskin, 1999; El-Sherif and Turitto, 2003, 2011), as well as the clinical evidence that magnesium sulfate is very effective for the treatment of TdP thus being considered the standard of care for this arrhythmia (Drew et al., 2010).

PPI-induced hypomagnesemia, for the first time described in 2006, has been increasingly recognized in the last years as a potentially life-threatening adverse event whose actual incidence is probably largely underestimated (Famularo et al., 2013). Two recent systematic reviews and meta-analysis, each one including nine studies and over 100,000 patients, consistently found that PPI users have a ~40–80% higher risk of developing hypomagnesemia when compared to non-users (Park et al., 2014; Cheungpasitporn et al., 2015).

PPI-associated hypomagnesemia occurs after extended treatments (>2 weeks, but in most cases > 1 year), is not clearly dose-related, and was reported with different PPIs, thus suggesting a class effect. Until PPI interruption, hypomagnesemia is refractory to oral or parenteral magnesium replacement irrespective of high-dose supplementation; when the PPI is stopped, serum magnesium levels returned to normal in less than 2 weeks (2011; Famularo et al., 2013). However, hypomagnesemia may recur after re-challenge with the same or a different PPI. In these patients, when prolonged antiacid treatment is needed, prescription of a H_2_ histamine receptor-blocker (H_2_-blocker) may be an appropriate therapeutic alternative (Famularo et al., 2013). In fact, although mechanisms of PPI-induced hypomagnesemia are not clear, hypochlorhydria does not seem to be involved. Pathogenesis possibly includes both gastrointestinal and renal losses, via dysfunction of the Transient Receptor Potential Melastatin 6/7 (TRPM6/7) located in the intestine as well as in the distal convoluted tubule (Famularo et al., 2013). Accordingly, recent data suggest that carriers of TRPM6 polymorphisms are at increased risk (Hess et al., 2017).

To date only three reports of patients who developed TdP while they were taking a PPI (i.e., omeprazole, pantoprazole, or lansoprazole, respectively) (Asajima et al., 2012; Bibawy et al., 2013; Hansen and Bruserud, 2016) have been described in the literature, in two cases associated with hypomagnesemia (Bibawy et al., 2013; Hansen and Bruserud, 2016). The results of the present study suggest that the phenomenon is significantly more common than reported, being probably underestimated because in the clinical practice PPIs do not currently receive the due attention as a factor potentially contributing to QTc prolongation and TdP. Consistently with literature data (Famularo et al., 2013), PPI-associated hypomagnesemia seems to be a class effect which requires extended drug administration to occur. In fact, although in our TdP patients most subjects used pantoprazole, hypomagnesemia was found to be associated with all 4 PPIs included in the AZCERT list (AZCERT, 2016) (i.e., pantoprazole, omeprazole, esomeprazole, lansoprazole), in all cases administered for an extended period of time (>2 weeks). Our data seem also to confirm that the risk of PPI-induced hypomagnesemia further increases when PPIs are co-administered with diuretics, probably as a result of an enhancement of the renal loss of magnesium. Conversely, although in PPI users hypomagnesemia has been reported to be often accompanied by hypocalcemia and hypokalaemia (Famularo et al., 2013), the prevalence of these electrolyte imbalances as well as serum calcium, potassium and sodium levels were similar in PPI+ vs. PPI− TdP patients, thus indicating a rather selective effect of this class of drugs on magnesium levels.

Another important suggestion arising from the present study is that PPI-associated changes in magnesium levels have a relevant clinical impact by increasing the risk of developing TdP in these patients. In fact, PPI users showed a significantly higher mean total number of QTc-prolonging risk factors per patient when compared to non-users. Nevertheless, despite a comprehensive evaluation also taking into account recently recognized “non-classical” QT-prolonging factors, such as anti-Ro/SSA antibodies (Yue et al., 2015; Lazzerini et al., 2016, 2017d) and systemic inflammatory activation (Lazzerini et al., 2015, 2017a,b), serum magnesium levels represented the only specific TdP risk factor which was significantly different between the two groups. Accordingly, when hypomagnesemia was excluded from the total risk factor count, this difference was no longer statistically significant.

Notably, we also found that magnesium levels in TdP/PPI+ patients were significantly lower when compared to C/PPI+ matched for age, gender and concomitant diseases. It suggests that TdP may act as a “clustering factor” for those patients, among the general population, who are more susceptible to the magnesium-lowering effect of PPIs, possibly as a result of a genetic predisposition (Hess et al., 2017). This view, further supporting the role of PPI-induced hypomagnesemia as a risk factor for TdP, warrants specific investigation.

Although our data point to the conclusion that PPIs can increase the risk of TdP by inducing hypomagnesemia, the involvement of additional, possibly molecule-related mechanisms could not be ruled out. In particular, this may be the case of lansoprazole which has been recently associated to an increased risk of QTc prolongation and TdP when used in combination with ceftriaxone, via direct blocking effects of the drug association on the hERG potassium channel (Lorberbaum et al., 2016; Lazzerini et al., 2017c). Indeed, 2 patients in our cohort were under current treatment with lansoprazole + ceftriaxone when TdP occurred, in 1 case in the absence of hypomagnesemia. Notably, it has been demonstrated that also lansoprazole alone significantly inhibits hERG potassium channel and related current IKr (−14%), although to a lesser extent when compared to the drug combination (−58%) (Lorberbaum et al., 2016). This may help explain why serum magnesium level was normal in one out of three case reports of PPI-associated TdP, in which lansoprazole administration precipitated arrhythmia development in a patients under long-term treatment with a drug known to directly prolong QTc (disopyramide) (Asajima et al., 2012). Thus, it cannot be ruled out that also in our patients, particularly those without hypomagnesemia, lansoprazole (and possibly also the other PPIs involved, since to date no specific patch-clamp studies are available) could have contributed to promote TdP occurrence also via a direct electrophysiological interference.

Our data suggest a number of important recommendations to translate in the clinical practice. In particular, patients may experience TdP in the presence of hypomagnesemia while they were under active treatment with a PPI. Such patients may be required to stop PPI treatment as it could have significantly contributed to development and maintenance of the electrolyte imbalance. Since it is expected that PPI-induced hypomagnesemia is refractory to magnesium oral or parenteral supplementation despite high doses (Famularo et al., 2013), drug discontinuation is a key action to normalize serum magnesium levels and thereby reduce the associated risk of TdP recurrence. This measure may be of particular importance in patients concomitantly requiring diuretic therapy, given the role of this class of drugs in exacerbating magnesium depletion. Moreover, based on the evidence that PPI-induced hypomagnesemia may rapidly recur after re-challenge with the same or a different PPI (median time ~2 weeks; Famularo et al., 2013), the alternative use of a H_2_-blocker may be appropriate in the case the patient needs prolonged antiacid treatment. Finally, since some data suggest that PPIs may also directly contribute to QTc prolongation via electrophysiological effects on the cardiomyocyte, it cannot be excluded that PPI discontinuation could be a useful therapeutic measure even in TdP patients without evidence of hypomagnesemia, particularly when the PPI involved is lansporazole and other known QT-prolonging drugs are concomitantly administered.

In conclusion, the present study demonstrates that PPI-induced hypomagnesemia is a more than expected common finding in unselected patients with TdP, significantly contributing to increase the cumulative risk of developing this life-threatening arrhythmia. Our real-life data provide important clinical evidence in support to AZCERT recommendations which cautiously already had warned about the potential role of PPI-induced hypomagnesemia in promoting TdP, despite only few cases were reported. Nevertheless, considering the relative small sample size as well as the main focus on magnesium levels, we did not perform any multivariate analysis on our population. Since this may represent a limitation of the study, larger sample studies are warranted to confirm our results. They should include non-TdP patients and/or younger populations, and could clarify whether PPIs significantly influence the QTc also regardless of hypomagnesemia.

In practice, more awareness is needed by the clinician when a PPI is prescribed since the safety profile of this class of drugs is probably not so neutral as commonly believed, specifically as regards the risk of life-threatening arrhythmias and SCD.

## Author contributions

PL: Conception and design of the work; PL, IB, FF, MA, MN, FV, BG, MC, and IL: Substantial contributions to the acquisition of data for the work; PL, RF, AG, MR, GC, FL-P, and PC: Substantial contributions to the analysis of data for the work; PL, RF, AG, MR, GC, FL-P, and PC: Substantial contributions to the interpretation of data for the work; PL and PC: Drafting the work; PL, RF, AG, MR, FL-P, and PC: Revising the draft of the work critically for important intellectual content; PL, IB, FF, MA, MN, FV, RF, AG, MR, BG, MC, IL, GC, FL-P, and PC: Final approval of the version to be published; PL, IB, FF, MA, MN, FV, RF, AG, MR, BG, MC, IL, GC, FL-P, and PC: Agreement to be accountable for all aspects of the work in ensuring that questions related to the accuracy or integrity of any part of the work are appropriately investigated and resolved.

### Conflict of interest statement

The authors declare that the research was conducted in the absence of any commercial or financial relationships that could be construed as a potential conflict of interest.

## Footnotes

^1^FDA Drug Safety Communication: Low magnesium levels can be associated with long-term use of proton pump inhibitor drugs (PPIs). Available at www.fda.gov/Drugs/DrugSafety/ucm245011.htm Accessed May 26 (2017).
