# Supplementary material for: Proton Pump Inhibitors and Serum Magnesium Levels in Patients With Torsades de Pointes
Source: Front Pharmacol. 2018 Apr 20;9:363. doi: 10.3389/fphar.2018.00363 (PMC5922007; doi:10.3389/fphar.2018.00363)
Supplement: Supplementary file 1 [file DataSheet1.docx]

**SUPPLEMENTARY MATERIAL**

**Supplementary Methods**

**Anti-Ro/SSA testing.**

Anti-Ro/SSA antibodies were determined by using different methods including: (i) fluoroenzyme-immunoassay(FEIA) by an EliA system (Phadia, Freiburg, Germany) using recombinant 60kD- and 52kD-Ro proteins, (ii) immuno-Western-blot analysis (iWB) using a kit with human Hep-2 cells as the source of extractive 60kD- and 52kD-Ro antigens, denatured by SDS (Marblot HEp-2; MarDx, Carlsbad,CA,USA), and (iii) line-blot immunoassay (LIA) by a kit using extractive 60kD-Ro protein purified (not denaturated) from bovine thymus and spleen by affinity chromatography, and recombinant 52kD-Ro proteins (Anti-ENA Profile Plus 1 Euroline, Euroimmun,Lübeck,Germany)*.* Moreover, all sera were also tested for anti-nuclear antibodies (ANA) by indirect immunofluorescence on human Hep-2000 cells(Fluorescent IgG ANA-Ro test, Immunoconcepts, Sacramento,CA,USA).
