# Supplementary material for: Proton Pump Inhibitors and Serum Magnesium Levels in Patients With Torsades de Pointes
Source: Front Pharmacol. 2018 Apr 20;9:363. doi: 10.3389/fphar.2018.00363 (PMC5922007; doi:10.3389/fphar.2018.00363)
Supplement: Supplementary file 2 [file DataSheet2.docx]

Supplementary table 1. Demographic, clinical and laboratory characteristics of control patients.

Patients,n 21

Age,median years (interquartile range) 84(76-86.5)

Females,n 14(67%)

Mean QTc,ms(range) 424.5±20.9(373-460)

Electrolyte imbalances,n 4/21(19%)

Hypokaliemia 2/21(10%)

Hypomagnesemia 2/21(10%)

Hypocalcemia 1/21(5%)

Concomitant diseases*,n 21/21(100%)

*Cardiac diseases* 21/21(100%)

Chronic coronary artery disease 8/21(38%)

Left ventricular hypertrophy 7/21(33%)

Dilated cardiomyopathy/heart failure 6/21(29%)

I-III degree atrioventricular block 3/21(14%)

Acute coronary syndrome 3/21(14%)

Sinus bradycardia 2/21(10%)

*Extra-cardiac diseases*  8/21(38%)

Chronic kidney disease 7/21(33%)

Diabetes mellitus type II 1/21(5%)

Subarachnoid haemorrhage 1/21(5%)

QTc prolonging-medications,n 10/21(48%)

Venlafaxine 3/21(14%)

Amiodarone 2/21(10%)

Paroxetine 1/21(5%)

Sertraline 1721(5%)

Citalopram 1/21(5%)

Ciprofloxacin 1/21(5%)

Promazine 1/21(5%)

Cetirizine 1/21(5%)

Mean medication number per patient 0.5±0.6

Anti-Ro/SSA positivity,n 3/21(14%)

Systemic inflammation,n† 14/21(67%)

C-reactive protein, mg/dl(range) 0.94(0.11-5.15)

Mean QTc-prolonging risk factor number per patient^§^ 3.4±1.4

_________________________________________________

Except where indicated otherwise, data are expressed as mean±standard deviation or median (range).

Appropriate serum potassium, calcium or magnesium measurements available in all 21 patients; anti-Ro/SSA antibodies tested in all 21 patients.

*Diseases recognized to be a risk factor for QTc prolongation. †Increased C-reactive protein level (>0.5 mg/dl) with or without a definite inflammatory disease. ^§^Including electrolyte imbalances, diseases, QTc-prolonging medications, anti-Ro/SSA positivity, and systemic inflammation.
