# Supplementary material for: Proton Pump Inhibitors and Serum Magnesium Levels in Patients With Torsades de Pointes
Source: Front Pharmacol. 2018 Apr 20;9:363. doi: 10.3389/fphar.2018.00363 (PMC5922007; doi:10.3389/fphar.2018.00363)
Supplement: Supplementary file 3 [file DataSheet3.docx]

Supplementary Table 2. Proton-pump inhibitors (PPIs) use in control patients.

Patients under active treatment with PPIs,n 12/21(57%)

Specific PPI used,n

Lansoprazole 6/12(50%)

Omeprazole 3/12(25%)

Pantoprazole 2/12(17%)

Esomeprazole 1/12(8%)

Treatment duration

Extended therapy (>2 weeks),n 10/12(83%)

Not extended therapy (<2 weeks),n 2/12(17%)

Daily dose,mg

Lansoprazole 17.5±6.1

Omeprazole 20.0±0

Pantoprazole 30.0±14.1

Esomeprazole 20

Route of administration,n

Oral 12/12(100%)

Intravenous 0/12(0%)

*_____________________________________*

Except where indicated otherwise, values are expressed as mean±standard deviation.
